# Acute Cholecystitis in Pregnancy: A Review of Incidence, Risk Factors, Diagnostic Challenges, Management, and Maternal–Fetal Outcomes

**DOI:** 10.3390/medicina62061087

**Published:** 2026-06-03

**Authors:** Zivile Sabonyte-Balsaitiene, Ugne Sleivyte, Jelena Volochovic, Augustas Beisa

**Affiliations:** 1 Clinic of Obstetrics and Gynecology, Institute of Clinical Medicine, Faculty of Medicine, Vilnius University, 01513 Vilnius, Lithuania; jelena.volochovic@santa.lt; 2 Faculty of Medicine, Vilnius University, 01513 Vilnius, Lithuania; usleivyte@gmail.com; 3 Clinic of Emergency Medicine, Institute of Clinical Medicine, Faculty of Medicine, Vilnius University, 01513 Vilnius, Lithuania; augustas.beisa@santa.lt

**Keywords:** pregnancy, acute cholecystitis, laparoscopic cholecystectomy

## Abstract

*Background and Objectives:* Acute cholecystitis occurs in approximately 0.3–0.6% of pregnant women and may pose significant risks to both the mother and the fetus. The condition is most commonly caused by gallstone-induced obstruction of the cystic duct, with pregnancy-related hormonal and physiological changes contributing to its development. The aim of this review was to summarize current evidence on the pathogenesis, diagnostic challenges, and management of acute cholecystitis during pregnancy, as well as to evaluate associated maternal and fetal outcomes. *Materials and Methods:* This study was conducted as a structured narrative review incorporating elements of a systematic literature search. A comprehensive search was performed in PubMed (MEDLINE), with additional sources identified through Google Scholar. Articles published between 2015 and 2025 were included. Eligible studies addressed pathogenesis, risk factors, diagnosis, management, or maternal and fetal outcomes. A total of 55 studies were included and analyzed qualitatively. *Results:* Acute cholecystitis during pregnancy presents diagnostic challenges due to nonspecific symptoms that may overlap with normal pregnancy-related conditions. Ultrasound remains the first-line imaging modality. Conservative management is associated with high recurrence and rehospitalization rates, as well as increased risks of adverse fetal outcomes, including preterm delivery, fetal growth restriction, and low birth weight. In contrast, laparoscopic cholecystectomy is associated with lower complication rates and improved outcomes, and can be safely performed during all trimesters when clinically indicated. *Conclusions:* Acute cholecystitis during pregnancy requires careful clinical evaluation and individualized management. Current evidence suggests that laparoscopic cholecystectomy is a safe and effective treatment option, and is widely considered the preferred approach in most cases. Conservative management may be appropriate in selected mild cases but is associated with a higher risk of recurrence and adverse outcomes.

## 1. Introduction

Hormonal and physiological changes occurring during pregnancy increase the risk of gallstone formation. Acute cholecystitis is a rare but potentially life-threatening condition in pregnant women [1]. It represents a medical, surgical, and obstetric emergency that may endanger both maternal and fetal health [2].

Gallstone disease is classified into asymptomatic, symptomatic, and complicated forms according to its clinical course [3]. In most cases, gallstones remain asymptomatic; however, they may lead to chronic inflammation of the gallbladder or obstruction of the cystic duct, resulting in acute cholecystitis [4]. During pregnancy, acute cholecystitis is most commonly caused by gallstone-induced obstruction of the cystic duct.

Diagnosis and management remain challenging due to nonspecific clinical manifestations and a broad range of differential diagnoses. Symptoms often overlap with normal pregnancy-related changes or other abdominal conditions, which may delay diagnosis and increase the risk of complications for both the mother and the fetus [1].

Currently, there is no universal consensus on the optimal management strategy. Although clinical guidelines increasingly recommend early laparoscopic cholecystectomy, conservative treatment has historically been preferred to avoid potential risks associated with surgery during pregnancy [5,6,7]. However, nonoperative management is associated with high rates of symptom recurrence and repeated hospitalizations during pregnancy and the postpartum period [6].

The available literature provides conflicting data regarding maternal and fetal outcomes associated with different treatment approaches, and high-quality prospective studies remain limited [8]. In recent years, advances in minimally invasive surgical techniques and anesthetic management have made surgical intervention safer during pregnancy [5,9].

The aim of this review is to provide a comprehensive overview of the diagnosis and management of acute cholecystitis during pregnancy, integrating current evidence and evaluating the clinical implications of different treatment strategies.

## 2. Materials and Methods

This study was conducted as a structured narrative review designed to summarize current evidence regarding acute cholecystitis during pregnancy. Although this review was not performed as a formal systematic review or meta-analysis, a structured literature search strategy and predefined eligibility criteria were applied to improve methodological transparency and reproducibility.

A literature search was conducted in PubMed (MEDLINE) and Google Scholar in January 2026. The search included articles published between January 2015 and January 2026.

The following search strategy was used in PubMed:

(“acute cholecystitis” AND pregnancy) OR (“cholelithiasis” AND pregnancy) OR (“laparoscopic cholecystectomy” AND pregnancy) OR (“gallstone disease” AND pregnancy) OR (“maternal outcomes” AND acute cholecystitis) OR (“fetal outcomes” AND acute cholecystitis).

Additional manual searches of reference lists from relevant articles were also performed to identify potentially eligible studies.

**Figure 1:**
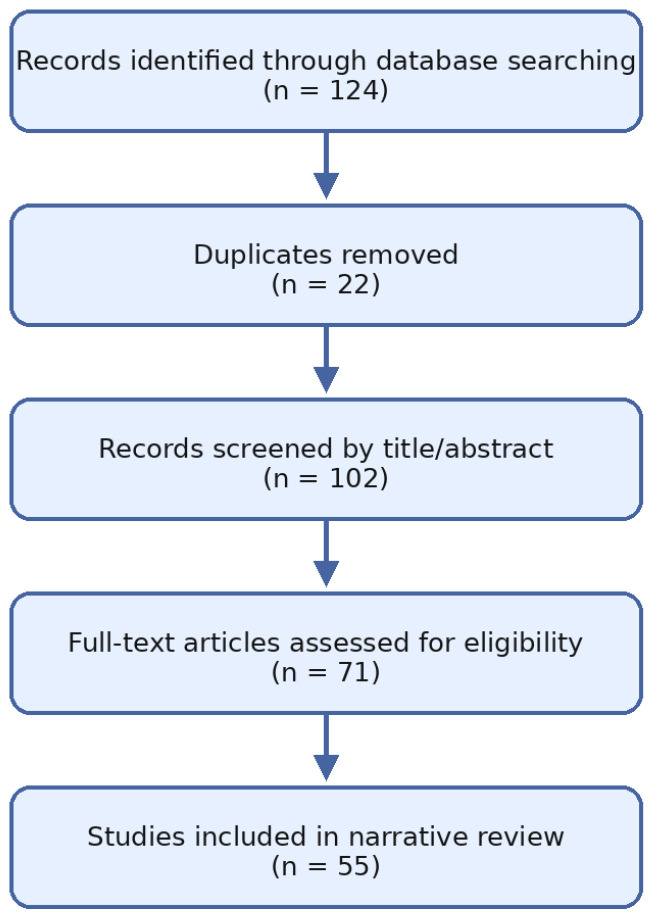
Simplified PRISMA-style flowchart.

The initial database search identified 124 records (PubMed *n* = 89; Google Scholar *n* = 35). After removal of duplicates, 102 records remained for title and abstract screening. Full-text assessment was performed for 71 articles, of which 55 studies were included in the final review. A simplified flowchart of the study selection process is presented in Figure 1. 

Studies were excluded because they were unrelated to pregnancy-associated acute cholecystitis, lacked sufficient clinical relevance, represented duplicate publications, or full text was unavailable.

Studies were eligible if they:

(1) Were published in English;
(2) Were available in full-text form;
(3) Focused on acute cholecystitis or gallstone-related disease during pregnancy;
(4) Addressed at least one of the following topics: epidemiology, pathogenesis, diagnosis, management, maternal outcomes, or fetal outcomes.

Non-English studies were excluded to ensure accurate interpretation of clinical data and methodological consistency across included publications.

Study selection and data extraction were performed independently by two reviewers. Any disagreements regarding study eligibility were resolved through discussion and consensus.

Due to the heterogeneous nature of the included evidence, which consisted of retrospective studies, observational studies, narrative reviews, clinical guidelines, and case reports, a formal quality assessment and quantitative meta-analysis were not performed. Instead, findings were synthesized descriptively and analyzed qualitatively.

## 3. Results

### 3.1. Prevalence

Cholelithiasis affects 10–15% of the general population and 20–40% of these patients develop various complications over time. The most common initial clinically apparent complication is acute calculous cholecystitis, accounting for about 10–15% of all symptomatic cases [10]. The disease is more prevalent in women than in men, with a female-to-male ratio of 2–6:1. This tendency is largely explained by the influence of female sex hormones and pregnancy-related physiological changes that promote gallstone formation [4,11].

The prevalence of gallstones among pregnant women ranges from 2% to 12%; however, only 0.05–0.3% of cases progress to acute cholecystitis [12,13]. Most gallstones remain asymptomatic, with asymptomatic cholelithiasis reported in up to 3.5% of pregnant women. Nevertheless, gallstones are responsible for approximately 90% of cases of acute cholecystitis during pregnancy [4]. The incidence may vary between countries, with higher rates reported in certain populations, such as in Saudi Arabia, where it can reach approximately 0.39%, possibly due to higher parity and genetic predisposition [1]. Reported prevalence and incidence rates vary considerably across studies and geographic regions. These differences may be related to variations in study populations, parity, dietary habits, obesity prevalence, genetic predisposition, diagnostic criteria, and study methodology.

Acute cholecystitis, which is defined as inflammation of the gallbladder wall, is most commonly caused by obstruction of the cystic duct by gallstones [14]. It is the second most common cause of acute abdominal pathology during pregnancy. The most common abdominal emergency is acute appendicitis [15]. Acute cholecystitis is one of the leading indications for non-obstetric surgery, occurring in approximately 1 in 1600 to 10,000 pregnancies [16]. The condition most frequently develops during the second and third trimesters, likely due to hormonal changes and reduced gallbladder motility. Despite these physiological alterations, the overall incidence of acute cholecystitis in pregnant women is not higher than in the nonpregnant population.

In most cases, gallstones during pregnancy remain asymptomatic; however, up to 90% of acute cholecystitis cases are associated with gallstones [4]. In rare instances, cholecystitis may occur in the absence of gallstones (acalculous cholecystitis), typically in the context of severe systemic infection, trauma, or prolonged fasting [14,17].

Acute cholecystitis during pregnancy may lead to serious complications, including suppurative inflammation, gallbladder perforation, cholangitis, and acute pancreatitis. In severe cases, septic shock may develop, posing a significant risk to both the mother and the fetus [18]. There is an increased risk of adverse pregnancy outcomes, such as miscarriage, intrauterine fetal death, stillbirth, and preterm birth [1,8].

### 3.2. Etiology and Pathogenesis

Bile is a complex biological fluid produced by hepatocytes. It performs two main physiological functions, the removal of insoluble metabolic products and participation in the digestive process, ensuring the emulsification of fats and the absorption of nutrients in the small intestine [13]. Bile is mainly composed of water (approximately 95%), bile acids, phospholipids (mostly phosphatidylcholine), and cholesterol, the balance of which is essential for maintaining its solubility and normal digestive function. Disruption of this balance plays a key role in gallstone formation. Enterohepatic circulation contributes to the regulation of bile composition by recycling bile acids, thereby maintaining cholesterol homeostasis and preventing excessive supersaturation [16].

Gallstone formation is a multifactorial process driven by three main mechanisms: supersaturation of bile with cholesterol, impaired gallbladder motility leading to bile stasis, and accelerated nucleation of cholesterol crystals [1,2,13]. These processes promote the aggregation and growth of microcrystals into macroscopic gallstones, which most commonly form in the gallbladder but may also migrate into the bile ducts. Based on their chemical composition gallstones are classified into cholesterol, pigment, and mixed types [13].

Hormonal and physiological changes during pregnancy play a significant role in increased risk of gallstone formation. These changes are primarily mediated by increased levels of estrogen and progesterone [19]. Elevated progesterone levels reduce gallbladder contractility by relaxing smooth muscle and decreasing responsiveness to cholecystokinin, resulting in impaired emptying and bile stasis [13]. At the same time, increased estrogen levels enhance hepatic cholesterol secretion into bile, contributing to cholesterol supersaturation [16,20].

The combined effect of these hormonal changes promotes bile stasis and cholesterol accumulation, creating favorable conditions for gallstone formation [21]. In addition to hormonal influences, other factors such as increased caloric intake, high-fat diet, insulin resistance, and possible alterations in immune response and gut microbiota may further contribute to this process [13].

Pregnancy is also associated with significant changes in bile composition. During the second and third trimesters, cholesterol concentration in bile increases, while the relative proportions of bile acids and phospholipids decrease, leading to bile supersaturation and the initiation of cholesterol crystallization. Alterations in enterohepatic circulation may further impair the bile acid balance and promote cholesterol secretion [16]. As a result, gallstones formed during pregnancy are predominantly of cholesterol origin, although pigment stones may also occur, particularly in women with higher parity [21,22].

### 3.3. Risk Factors

Gallstone formation is a multifactorial process, influenced by both genetic (hereditary) and exogenous (environmental and lifestyle) factors [13]. In pregnant women, the main risk factors for cholelithiasis and cholecystitis include hormonal changes, multiparity, advanced maternal age, increased body mass index (BMI), excessive weight gain during pregnancy, physical inactivity, unhealthy diet, previous history of cholelithiasis, and use of oral contraceptives [20,23]. Multiparity is considered one of the most significant risk factors, as the risk of gallstone disease increases with the number of pregnancies. Women who have had four or more pregnancies have been shown to have an approximately 14% higher risk of developing gallbladder disease compared to those with fewer pregnancies [20,21]. This association is likely related to repeated hormonal and physiological changes, including alterations in gallbladder motility and bile composition. However, some studies report conflicting results, suggesting that pregnancy itself may not be an independent risk factor [20]. Advanced maternal age also increases the risk due to the cumulative effect of multiple predisposing factors over time [13]. Similarly, increased BMI before pregnancy and excessive weight gain during pregnancy are important contributors, as they are associated with increased insulin resistance, enhanced hepatic cholesterol secretion, and impaired gallbladder motility, promoting bile stasis and gallstone formation [21]. Dietary habits also play an important role—a high-calorie, high-carbohydrate, low-fiber diet is considered a predisposing factor [13].

### 3.4. Symptoms

Clinical symptoms of acute cholecystitis during pregnancy are generally similar to those in the general population [1]. The disease usually begins with sudden, persistent abdominal pain localized to the right upper quadrant or epigastrium, lasting more than 4–6 h, and potentially radiating to the right shoulder or back [20,21]. Dyspepsia and fat intolerance are present in almost all cases [4]. Approximately half of patients experience nausea and vomiting, and as many as 70–80% report a history of gallstones or recurrent episodes of biliary colic.

Physical examination findings vary depending on the severity of the cholecystitis. Fever is present in approximately 30% of cases, and right upper quadrant tenderness with guarding may be observed, whereas a positive Murphy’s sign is less frequently detected [1,6]. Although Murphy’s sign is commonly positive in nonpregnant patients, it is considered less reliable during pregnancy, particularly in the third trimester [4]. Other symptoms may include general signs of inflammation or intoxication [1,6].

However, it is important to note that symptoms such as nausea, vomiting, and abdominal pain are also common during normal pregnancy, which may delay the diagnosis of acute cholecystitis [15]. These symptoms may overlap with several pregnancy-related and abdominal conditions, potentially delaying the diagnosis of acute cholecystitis. A differential diagnosis should include HELLP syndrome, acute fatty liver of pregnancy, acute appendicitis, acute pancreatitis, hepatitis, pyelonephritis, peptic ulcer disease, and other hepatobiliary disorders. HELLP syndrome and acute fatty liver of pregnancy may present with right upper quadrant pain and abnormal liver function tests, whereas pancreatitis may be associated with elevated pancreatic enzymes and more severe epigastric pain. Therefore, careful clinical evaluation combined with laboratory and imaging studies is essential to establish an accurate diagnosis and avoid delays in management.

### 3.5. Maternal and Fetal Outcomes

Inadequately managed acute cholecystitis during pregnancy is associated with an increased risk of adverse maternal and fetal outcomes [24,25]. Conservative management has been consistently linked to high recurrence and rehospitalization rates, ranging from 58% to 78.5%. Hassanesfahani et al. (2025) reported a recurrence rate of 77%, with up to 39% of patients requiring rehospitalization [21]. Similarly, Barut et al. (2019) [6] observed rehospitalization rates of 78.5%, longer hospital stays (11 vs. 3 days compared to surgical management), and recurrence in 84.6% of cases [6], while Zhang et al. (2023) reported a rehospitalization rate of 58.06% with conservative treatment [18].

Conservative management is additionally associated with unfavorable fetal outcomes: preterm delivery (5.9–28.5%), fetal growth restriction (7.3%), and low birth weight (up to 8.5%) [1,6,18,21,26,27]. Mehmet İlhan et al. (2016) reported maternal mortality of 1.7% alongside fetal complications such as spontaneous abortion (1.7%), low birth weight (8.5%), and preterm birth (6.8%) [26]. These findings suggest that, despite avoiding operative risks, conservative treatment carries a substantial burden of recurrence and complications.

Surgical management—particularly laparoscopic cholecystectomy—demonstrates more favorable outcomes. Nasioudis et al. (2016) reported low maternal and fetal complication rates, including bile leakage (1.84%) and hypotension (1.47%), as well as low fetal complication rates, such as preterm delivery (5.7%), fetal loss (0.4%), and intraoperative fetal bradycardia (0.17%) [27]. A large population-based study by Hantouli et al. (2024) showed that acute cholecystitis increases the risk of adverse pregnancy outcomes (OR 1.69), whereas surgical treatment significantly reduces this risk compared to nonoperative management (OR 0.75), regardless of trimester [8]. Martins et al. (2025) further demonstrated that surgical management reduced adverse pregnancy outcomes (OR 0.60; 95% CI 0.42–0.87) and shortened hospital stay (mean difference—7.15 days; 95% CI—7.83 to 6.47), while no significant differences were observed in maternal mortality, pregnancy loss, preterm delivery, and readmission rate [9]. Similarly, Iyer et al. (2026) confirmed that surgical intervention can be safely performed in all trimesters, with no significant differences in maternal and fetal outcomes, including pre-eclampsia, pregnancy loss, or preterm delivery [28].

Overall, current evidence indicates that conservative management is associated with higher recurrence, increased hospitalization, and worse fetal outcomes, whereas laparoscopic cholecystectomy appears to be a safe and effective treatment option during pregnancy.

Emerging evidence also suggests that maternal biliary disease may be associated with long-term hepatopancreatobiliary morbidity in offspring; however, these findings are not specific to acute cholecystitis and require further investigation [29].

### 3.6. Diagnostics

Physiological and anatomical changes during pregnancy complicate the recognition and diagnosis of acute cholecystitis. Symptoms often overlap with normal pregnancy symptoms or other pregnancy-related or abdominal conditions, which may delay diagnosis and increase the risk of complications for both the mother and the fetus. Therefore, accurate diagnosis requires a comprehensive approach combining clinical evaluation, laboratory testing, and radiological studies [24].

There are no specific laboratory biomarkers to confirm the diagnosis of acute cholecystitis. In suspected cases, pregnant women should undergo clinical assessment along with laboratory testing, which should include a complete blood count and biochemical tests evaluating liver and pancreatic function (AST, ALT, GGT, ALP, bilirubin, amylase, and lipase) [30].

Leukocytosis with neutrophilia and elevated C-reactive protein levels may indicate an inflammatory process. These findings should be interpreted with caution, as physiological leukocytosis is common during pregnancy, particularly in the third trimester, where the leukocyte count can reach up to 20,000 cells/mL. Some studies suggest that CRP levels above 40 IU/L may be more commonly associated with bacterial infection; however, CRP interpretation during pregnancy remains nonspecific and should not be used as an isolated diagnostic marker [1]. Liver function abnormalities may suggest biliary obstruction, while elevated lipase levels are important in excluding acute pancreatitis. Overall, laboratory findings are nonspecific and should always be interpreted in the context of clinical presentation and imaging studies [4,15,30].

Imaging plays a crucial role in the diagnosis of acute cholecystitis. Both ultrasound and magnetic resonance imaging can be performed for the initial diagnostic examination [31]. Ultrasound is the first-line method due to its availability, safety, and absence of ionizing radiation. It is a fast and economical method, too. Its sensitivity ranges from 50% to 88% and specificity from 80% to 88% [32]. The diagnostic accuracy of ultrasound may be limited by operator experience, increased body mass index, bowel gas, and anatomical changes during pregnancy, particularly in the second and third trimesters. Image quality can be improved by applying certain techniques, such as changing the patient’s position (e.g., lying on the left side or standing) and holding the breath, which help optimize the visibility of the gallbladder. Typical findings include gallbladder wall thickening (≥5 mm), pericholecystic fluid, gallstones, and a sonographic Murphy’s sign [1,32]. Additional findings may include gallbladder dilation, visualization of stones, or the presence of air bubbles in the gallbladder.

When ultrasound findings are inconclusive, magnetic resonance imaging (MRI), including magnetic resonance cholangiopancreatography (MRCP), is recommended as a second-line imaging modality. MRI provides a comprehensive evaluation of abdominal structures without ionizing radiation and is generally considered safe during pregnancy. Current evidence suggests that non-contrast MRI is not associated with an increased risk of fetal malformations, hearing impairment, or adverse childhood outcomes [32,33]. However, MRI during the first trimester should be performed only when clinically indicated, as data regarding early fetal exposure remain limited [33]. MRI is particularly useful for detecting bile duct stones and differentiating biliary pathology from other pregnancy-related conditions. Gadolinium-based contrast agents cross the placenta and have been associated with an increased risk of rheumatological, inflammatory, or infiltrative skin disorders, as well as stillbirth or neonatal death; therefore, they should generally be avoided during pregnancy and used only in exceptional cases when the diagnostic benefit clearly outweighs the potential fetal risk [33,34]. Imaging considerations may also vary according to gestational trimester, as the visualization of abdominal structures may become increasingly limited during late pregnancy because of the enlarged uterus and altered anatomical relationships.

Abdominal and pelvic computed tomography (CT) is not recommended during pregnancy due to its lower sensitivity and specificity, and the risk of ionizing radiation to the fetus [1]. It may be considered in selected cases when other imaging modalities are inconclusive and the potential diagnostic benefit outweighs the risks. In such cases, radiation exposure should be minimized according to the ALARA principle (“as low as reasonably achievable”). According to the American College of Obstetricians and Gynecologists (ACOG), if CT is necessary for diagnosis, is complementary to ultrasound or MRI, or is more readily available in a particular clinical situation, it should not be avoided in pregnant women. Although iodinated contrast media crosses the placenta and enters the fetal circulation or amniotic fluid, it has not been shown to cause mutagenic or teratogenic effects or to adversely affect the fetal thyroid gland [34].

### 3.7. Treatment

According to current clinical guidelines, the management of acute cholecystitis during pregnancy should follow a structured and individualized approach. Treatment strategies are divided into conservative and surgical management.

Management should be individualized, taking into account the condition of the mother and fetus, gestational age, disease severity and duration, comorbidities, and other associated medical conditions.

Although Tokyo guidelines for the management of acute cholecystitis are not specifically intended for pregnant women, they may still be useful in selecting the most appropriate treatment approach in individual cases [35].

The conservative approach remains commonly used in clinical practice and includes hospitalization, fasting, intravenous fluid therapy, analgesia, and antibiotics [14]. Second- and third-generation cephalosporins are most frequently used, as they are considered safe during all trimesters of pregnancy [1]. Paracetamol and opioid analgesics are preferred for pain control, while nonsteroidal anti-inflammatory drugs are generally avoided due to potential fetal risks [19]. Conservative treatment may be effective during the initial hospitalization, particularly in mild cases. However, symptoms cannot be effectively controlled in approximately 27–36% of patients, and persistent abdominal pain and vomiting may lead to uterine contractions, intrauterine fetal distress, and an increased risk of miscarriage or preterm delivery [6].

It is important to distinguish uncomplicated biliary colic from confirmed acute cholecystitis, as the natural course and recurrence risk differ substantially between these conditions. While biliary colic may often be managed conservatively with a lower complication risk, acute cholecystitis is associated with higher rates of recurrence, rehospitalization, and adverse maternal–fetal outcomes, particularly when definitive surgical treatment is delayed. Conservative management is also associated with a high rate of symptom recurrence and rehospitalization. Studies report recurrence rates of up to 70%, with a substantial proportion of patients requiring repeated hospitalization or subsequent surgical intervention [36,37,38]. Similarly, Hassanesfahani et al. (2025) reported that up to 77% of patients managed conservatively experienced symptom recurrence, with many ultimately requiring surgical intervention in the postpartum period, further highlighting the limitations of nonoperative management [21]. Zhang et al. (2023), in a multicenter retrospective study of third-trimester patients, reported significantly lower readmission rates in the surgical group (9.1%) compared to conservative treatment (58.06%), along with shorter hospital stay, highlighting the limitations of conservative management and the potential benefit of timely intervention [18]. Furthermore, Hantouli et al. (2024) demonstrated that nonoperative management is associated with higher odds of adverse pregnancy outcomes compared to surgical treatment, highlighting the limitations of conservative therapy [8]. Additionally, a large nationwide analysis by Rios-Diaz et al. (2020) showed that nonoperative management is associated with a threefold increase in maternal–fetal complications (OR 3.0) and higher readmission rates, further underscoring the limitations of conservative treatment [39]. Despite guideline recommendations favoring surgical management, operative intervention remains underutilized in clinical practice. Weaver et al. (2025) reported that only 27.0–34.9% of pregnant patients with cholecystitis undergo intervention, highlighting a persistent gap between evidence-based recommendations and real-world management [40]. Moreover, Cheng et al. (2021) reported that laparoscopic cholecystectomy is associated with significantly lower rates of adverse fetal outcomes compared to nonoperative management (OR 0.41) and that delays in surgical intervention further increase the risk of complications [36].

Surgical management primarily includes laparoscopic cholecystectomy, which is increasingly recommended as the preferred approach. Current evidence suggests that laparoscopic cholecystectomy can be safely performed during all trimesters of pregnancy and is associated with lower rates of recurrence, rehospitalization, and complications compared to conservative management [5,40,41]. Advances in minimally invasive techniques, anesthetic management, and surgical expertise have significantly expanded the use of laparoscopy in pregnant patients. Some concerns remain regarding potential uterine injury and the effects of pneumoperitoneum on fetal well-being. Current evidence indicates that laparoscopic cholecystectomy can be safely performed in all trimesters when clinically indicated. International guidelines differ slightly in recommendations, with some favoring second-trimester intervention, while others support surgery regardless of gestational age when necessary [42]. Intraoperative precautions include patient positioning to avoid vena cava compression, use of low intra-abdominal pressure, and careful trocar placement to minimize the risk of uterine injury. Maternal respiratory parameters should be closely monitored, and fetal assessment is recommended before and after surgery [43].

Multiple studies have demonstrated low rates of maternal and fetal complications associated with laparoscopic cholecystectomy, supporting its safety across all trimesters [7,12].

Other surgical management methods, including gallbladder drainage and open surgery, are reserved for selected cases.

In patients who are not suitable candidates for laparoscopic cholecystectomy, gallbladder drainage may be used as a temporizing measure. Percutaneous transhepatic cholecystostomy is the most commonly used technique, demonstrating high clinical success rates but also associated with complications such as infection, catheter-related issues, recurrence and more difficult and complicated delayed operation.

Open cholecystectomy should be reserved for cases where laparoscopic surgery is contraindicated or technically not possible, such as generalized peritonitis [5,44]. Open procedures are associated with greater surgical trauma, longer recovery time, and a higher risk of complications, including thromboembolic events and wound infections. Evidence suggests worse maternal and fetal outcomes and prolonged hospitalization following laparotomy [2,45].

Endoscopic approaches, including transpapillary and endoscopic ultrasound-guided drainage, have been developed as alternatives. Their use during pregnancy remains limited and is typically restricted to specialized centers. These methods may serve as temporary strategies until definitive surgical treatment can be safely performed [44,45].

Overall, while conservative management may be appropriate in selected mild cases, early surgical intervention is increasingly favored due to its lower recurrence rates and more favorable long-term outcomes. The summary of key studies comparing conservative and surgical management are reported in Table 1.

### 3.8. Limitations of Current Evidence

The currently available evidence regarding acute cholecystitis during pregnancy remains limited by several important factors. Most studies are retrospective and observational in design, which introduces risks of selection bias, incomplete data collection, and confounding factors. In addition, substantial heterogeneity exists between studies regarding patient populations, diagnostic criteria, disease severity, treatment strategies, and reported maternal and fetal outcomes, limiting direct comparison of findings.

Potential selection bias should also be considered when comparing operative and conservative management groups, as treatment decisions are frequently individualized according to gestational age, disease severity, maternal clinical status, and institutional expertise. Furthermore, trimester-specific differences may influence both management decisions and outcomes, since surgical intervention during late pregnancy may be technically more difficult, whereas first-trimester intervention may raise concerns regarding fetal safety. Recent large population-based data demonstrated that cholecystectomy during pregnancy was associated with lower odds of adverse pregnancy outcomes across all trimesters, with the greatest benefit observed during the third trimester [8]. These findings further support current guideline recommendations favoring surgical management when clinically indicated. High-quality prospective studies are still needed to establish more definitive management recommendations.

## 4. Conclusions

In the management of acute cholecystitis during pregnancy, a careful balance between the risks of surgical intervention and the potential complications of disease progression is essential [46]. Current evidence indicates that conservative treatment is associated with a high risk of recurrence and repeated hospitalizations, suggesting that it is often a temporary rather than definitive solution [6,18]. Laparoscopic cholecystectomy is widely considered the preferred treatment option, demonstrating favorable maternal and fetal outcomes, as well as lower complication and recurrence rates compared to conservative management [47]. Despite its advantages, surgical intervention may be technically challenging, particularly in late pregnancy, and should be individualized based on gestational age, disease severity, and patient-related factors [48,49]. In patients who are not suitable candidates for laparoscopic cholecystectomy, gallbladder drainage may be used as a temporizing measure. Open cholecystectomy remains reserved for complicated cases when laparoscopic surgery is not feasible, although it is associated with higher morbidity [43,50]. Overall, timely surgical management, when indicated, appears to provide the most effective and durable treatment, while conservative approaches may be appropriate in selected mild cases.

## Figures and Tables

**Table 1:** Summary of key studies comparing conservative and surgical management.

Study	Year	Design	Key Findings
Mahjoubi et al. [1]	2023	Retrospective study	Open surgery was associated with higher complication rates compared with laparoscopic management
Barut et al. [6]	2019	Retrospective study	Conservative management was associated with high recurrence and rehospitalization rates, longer hospital stays, and increased risk of preterm delivery
Hantouli et al. [8]	2024	Population-based retrospective cohort study	Cholecystectomy during pregnancy was associated with lower odds of adverse pregnancy outcomes across all trimesters compared with nonoperative management, with the greatest benefit observed during the third trimester
Martins et al. [9]	2025	Systematic review and meta-analysis	Surgical management was associated with reduced adverse pregnancy outcomes and shorter hospital stay compared with conservative treatment
Zhang et al. [18]	2023	Multicenter retrospective study	Conservative management was associated with increased readmission rates and longer hospital stay compared with surgical treatment
Hassanesfahani et al. [21]	2025	Retrospective observational study	Nonoperative management remained the predominant approach despite guideline recommendations and was associated with high recurrence and readmission rates
İlhan et al. [26]	2016	Retrospective study	Conservative management was associated with adverse maternal and fetal outcomes, including low birth weight and preterm delivery
Nasioudis et al. [27]	2016	Systematic review	Laparoscopic cholecystectomy demonstrated low rates of maternal and fetal complications
Iyer et al. [28]	2026	Retrospective cohort study	Conservative management was associated with increased rates of cesarean delivery, preterm birth, and neonatal intensive care unit admission
Rios-Diaz et al. [39]	2020	Nationwide retrospective analysis	Nonoperative management was associated with higher rates of maternal–fetal complications, cesarean delivery, and fetal growth restriction

## Data Availability

No new data were generated or analyzed in this study.

## Abbreviations

The following abbreviations are used in this manuscript:

BMI	body mass index
CT	computed tomography
CRP	C-reactive protein
GGT	gamma-glutamyl transferase
MRI	magnetic resonance imaging
MRCP	magnetic resonance cholangiopancreatography
NICU	neonatal intensive care unit
OR	odds ratio
CI	confidence interval
ALARA	as low as reasonably achievable
ACOG	American College of Obstetricians and Gynecologists
